# Motive-oriented therapeutic relationship building for patients diagnosed with schizophrenia

**DOI:** 10.3389/fpsyg.2015.01294

**Published:** 2015-09-02

**Authors:** Stefan Westermann, Marialuisa Cavelti, Eva Heibach, Franz Caspar

**Affiliations:** ^1^Department of Clinical Psychology and Psychotherapy, Institute of Psychology, University of Bern, Bern, Switzerland; ^2^Translational Research Center, University Hospital for Psychiatry and Psychotherapy, Bern, Switzerland; ^3^Private Psychotherapeutic Practice Heibach, Lastrup, Germany

**Keywords:** schizophrenia, motive-oriented therapeutic relationship, Plan Analysis, case conceptualization, therapeutic relationship, psychosis, CBT

## Abstract

Treatment options for patients with schizophrenia demand further improvement. One way to achieve this improvement is the translation of findings from basic research into new specific interventions. Beyond that, addressing the therapy relationship has the potential to enhance both pharmacological and non-pharmacological treatments. This paper introduces motive-oriented therapeutic relationship (MOTR) building for schizophrenia. MOTR enables therapists to proactively adapt to their patient’s needs and to prevent problematic behaviors. For example, a patient might consider medication as helpful in principle, but the rejection of medication might be one of his few remaining means for his acceptable motive to stay autonomous despite hospitalization. A therapist who is motive-oriented proactively offers many degrees of freedom to this patient in order to satisfy his need for autonomy and to weaken the motivational basis for not taking medication. MOTR makes use of findings from basic and psychotherapy research and is generic in this respect, but at the same time guides therapeutic action precisely and flexibly in a patient oriented way.

## The Therapeutic Relationship—A Starting Point for Improving Schizophrenia Treatments?

Besides antipsychotic medication, cognitive behavioral therapy for psychosis (CBTp) is an evidence-based treatment option for patients with schizophrenia and related disorders (Pfammatter et al., 2006; Wykes et al., 2008; Turner et al., 2014). Even though CBTp and antipsychotic medication are effective, their effects are only medium-sized, and not every patient profits. Meta-analyses report medium effect-sizes both for second generation antipsychotics compared to placebo (Hedge’s *g* = 0.51; Leucht et al., 2008) and for CBTp compared to social support (*g* = 0.42; Turner et al., 2014). Thus, there is room for further improvement of pharmacological and psychotherapeutic treatment options for patients with schizophrenia. Considerable efforts are being made to translate findings from basic research in order to improve CBTp (Freeman and Garety, 2014). For example, interventions for insomnia, worrying or trauma have been adapted for schizophrenia treatment (Jackson et al., 2009; Myers et al., 2011; Freeman et al., 2015). Apart from more and more specific interventions, are there other targets for improving the treatment of schizophrenia?

The relation of therapeutic alliance and outcome is a robust finding in psychotherapy research (Flückiger et al., 2012). In the treatment of schizophrenia, direct evidence of the alliance-outcome relation is scarce but consistently positive for psychotherapy (Svensson and Hansson, 1999; Priebe et al., 2011; Huddy et al., 2012), for case management (Farrelly et al., 2014) and also for compliance with pharmacotherapy (Lacro et al., 2002). Thus, the therapeutic relationship is an important factor in the treatment of schizophrenia and a potential target for improving both pharmacological and psychological interventions.

In stark contrast to the elaborated models of the development and maintenance of psychotic symptoms (e.g., Bentall et al., 2001; Garety et al., 2001), there is no evidence-based theoretical framework of the therapeutic relationship in schizophrenia treatment that informs therapeutic action. Consequently, research on the therapy relationship in schizophrenia treatment is theory-driven only to a minor degree and focuses on patient (e.g., symptoms, insight into illness, attachment style; Kvrgic et al., 2013) and therapist variables (e.g., empathy or trustworthiness; Evans-Jones et al., 2009), or investigates the potentially negative impact of specific intervention strategies, such as cognitive dispute, on therapeutic relationship (Wittorf et al., 2010). Many authors agree that therapeutic relationship building is important and challenging in CBTp (Dilks et al., 2013; Johansen et al., 2013; Jung et al., 2014), but the recommendations are divergent and range from specific suggestions for difficult situations in therapy (e.g., mistrust or affective flattening; Klingberg and Wittorf, 2012) to empathy and mindfulness trainings in therapist qualification programs (Jung et al., 2014). In addition, treatment manuals for schizophrenia highlight the importance of building and maintaining a good therapeutic relationship (e.g., Lincoln, 2006; Chadwick, 2008) and recommend specific therapeutic techniques (e.g., emotional validation and normalization; Lincoln, 2006) and stances (e.g., radical collaboration; Chadwick, 2008). However, empirical findings and practical recommendations are not integrated within an overarching framework. Nonetheless, a growing number of psychotherapy approaches focus on the therapy relationship in the treatment of patients with schizophrenia (e.g., metacognitive interpersonal therapy, Salvatore et al., 2012; for a case study, see Hillis et al., 2015; as well as a mentalization-based approach, Brent, 2015).

In sum, the therapeutic relationship is a promising starting point for improving both pharmacological and psychotherapeutic schizophrenia treatments, but there is need for a therapy relationship framework in schizophrenia treatment. A framework for building and maintaining a therapeutic relationship in schizophrenia treatment has to meet multiple demands. An optimal framework (a) integrates existing empirical findings on the therapy relationship, (b) informs therapeutic action both proactively and reactively, individualized for each patient, (c) makes full use of findings from basic research, and (d) is compatible with the diversity of interventions (e.g., pharmacological and psychotherapeutic interventions). Motive-oriented therapeutic relationship (MOTR; Caspar, 2011) is a framework that has the potential to meet these requirements and has begun to demonstrate its utility in mental disorders such as borderline personality disorder (Kramer et al., 2011) and narcissistic personality disorder (Kramer et al., 2014). MOTR was also applied to severe Axis-I disorders such as bipolar disorder (Kramer et al., 2009). Moreover, a flexible, motive-oriented therapist behavior seems to be particularly beneficial for patients with more severe symptomatology and less resources (Grawe et al., 1990).

## Motive-Oriented Therapeutic Relationship Building in Schizophrenia Treatments

What is MOTR and how does it work? In pharmacological and psychotherapeutic treatments, patient behavior which interferes with a generally useful therapeutic procedure and potentially at the end with outcome can be defined as *problematic behavior*. In that sense, refusing medication, not acknowledging a mental disorder or concealing symptoms are problematic behaviors in the treatment of schizophrenia. Therapists who insist on taking medication, try to argue patients into being insightful or try to convince patients of giving up their delusional beliefs are trying to deal with problematic behavior. However, might such a therapist behavior also be problematic? MOTR helps the therapist to address problematic patient behavior in an unproblematic, adaptive way. In this section, MOTR is introduced and its application to schizophrenia treatment will be illustrated in three exemplary domains (medication compliance, delusional beliefs, and negative symptoms).

A central tenet of MOTR building is that each problematic behavior of a patient has at least one unproblematic, acceptable superordinate purpose or motive (Caspar, 2011). If therapists have an idea of the superordinate motive of a problematic behavior (i.e., its instrumentality), they have an increased chance to proactively address this acceptable purpose without reinforcing the problematic behavior. When looking for an unproblematic motive one infers motives up in an instrumental hierarchy (in which concrete instrumental behaviors are on the bottom), and once one has arrived at a higher level asks “Is this motive unproblematic?”. If the motive is still problematic, one keeps asking “And to which superordinate motives does this motive serve?” until one reaches an unproblematic motive. But as motives become less and less specific when approaching the level of general needs, one does not go higher than necessary to avoid spoiling resources by lacking correspondence to the individual motives of a particular patient. For example, a patient might reject medication. An acceptable superordinate motive could be to experience oneself as being autonomous. Thus, one can look for complementary therapist behaviors such as proactively offering many degrees of freedom in therapy sessions. If the superordinate motives are saturated or even oversaturated by a therapist independent of the problematic means (patient experiences himself as autonomous), the problematic behavior is not needed anymore by the patient (rejection of medication in order to increase autonomy no longer necessary) and by being proactive and non-contingent in time to patient behavior, the therapist does not (unintentionally) reinforce the problematic behavior, which might happen when reacting on a behavioral level (forcing to take medication).

In addition to reducing problematic patient behavior, MOTR aids the therapist to satisfy patient’s basic needs by being responsive to motives and behaviors, which are acceptable from the outset. Revealing the individually most important topics and motives, a case conceptualization also leads the way to complementary offers in the most valuable currency for this patient, and helps to prevent spoiling resources by attempting to serve all needs in an undifferentiated way. For example, when a patient has a pronounced need for orientation and control, the issue of being an independent decision-maker may be of particular importance for this patient, and is seen as unproblematic motive, the therapist creates situations in which the patient can experience himself as decision-maker.

### CBTp Techniques for Relationship Building From a MOTR Perspective

Currently, the primary CBTp techniques for building and maintaining the therapy relationship are normalizing and (emotional) validating (e.g., Lincoln, 2006). From a MOTR perspective, both techniques are motive-oriented in many but not all therapy situations. Normalizing conveys to the patient that many other people have similar experiences. First, this is likely to satisfy the need for affiliation, as one is part of a group of many similar human beings. Second, normalizing implies that one is not “crazy” or “abnormal,” preventing further threats to self-esteem and also preventing a threat for autonomy (“I’m crazy and will be in a ward for the rest of my life”). Last not least, normalizing may help to satisfy the need for orientation and control, because the information that even psychotic symptoms are rather normal in fact provides orientation. With regard to validation, similar motive-oriented consequences for patients are likely. Particularly, validation means that patient and therapist do not argue about the truth of a delusional belief or the authenticity of voices. In contrast, the therapist understands the emotional and behavioral reactions of the patient and communicates this understanding and empathy. This is a corrective experience for many patients as they are no longer forced to defend their view of the world or conceal it in order to protect their self-esteem and need for orientation. In line with this, the effect of normalizing and validation compared to educating supported psychological treatment motivation in an analog study (Lüllmann and Lincoln, 2013). However, if the maintenance of a delusional belief of a patient has a strong instrumentality for self-esteem (e.g., grandiose or erotomanic delusions, voice of God, etc.), a standard normalizing approach would be adverse according to MOTR because it endangers an important instrumentality of the behavior (e.g., a therapist saying “Many people think that they have a special connection to God, this is normal” is likely to threaten the need for self-esteem enhancement and/or attachment of a patient).

### Medication Compliance

The behavior “refuses medication” might primarily serve to satisfy the need for autonomy, as in the example in the previous section. However, another patient might refuse medication in order to evoke additional sessions with a therapist or a closer contact with caregivers, to satisfy his need for attachment. Yet, another patient who beliefs he is persecuted outside the ward might refuse medication to prolong his hospitalization, in order to satisfy his need for security. Furthermore, another patient might “forget” taking medication in order to avoid being reminded of being ill, which in turn protects self-esteem. And yet another patient may simply want to avoid negative side effects. Thus, one problematic behavior can serve very different motivational purposes. In addition, a problematic behavior can be multiply determined (e.g., serving autonomy *and* affiliation).

MOTR suggests different therapeutic stances and interventions, depending on the instrumentality of the problematic behavior. With regard to autonomy and control, satisfying the Plan “exercise control” by broadening the opportunity for decision-making in other domains can be expected to weaken the motivational basis for not taking medication. In contrast, a dispute of the pros and cons of medication each time the patient rejects medication would be a positive reinforcement of the patient behavior (C+), because he or she experiences herself as in control which would be in line with the basic need (given that the medication is not forced). When the problem behavior is instrumental for attachment (evoking more caregiver contact by refusing medication), regular and non-contingent short contacts would be derived from the MOTR approach in order to saturate the motivational basis for rejecting mediation.

### Maintenance of a Delusional Belief

In line with the cognitive model of psychosis (Garety et al., 2001), the maintenance of a delusional belief is likely to satisfy the need for orientation and control in many patients. In addition, the direct cognitive disputation of such a belief can be seen as threat for the self-esteem. In that sense, “typical” therapeutic strategies such as psychoeducation or persuading patient into being insightful are problematic therapist behaviors for many patients, as they pose a direct threat to the self-esteem and to the need for orientation and control. However, if a patient has a first episode, no elaborated delusional beliefs and a deprived need for orientation and control, then psychoeducation and the facilitation of a biopsychosocial problem model is expected to be very helpful, according to MOTR. Thus, MOTR enables the therapist to make informed decisions when to use which intervention.

If the maintenance of a delusional belief is multiply determined (i.e., is instrumental for two or more superordinate motives) and has an instrumentality for self-esteem (e.g., grandiose delusion), MOTR would suggest that the therapist supports the self-esteem in each session. In such a way, MOTR would satisfy the self-esteem motive independent of grandiosity delusion thus paving the way for direct interventions regarding the delusional belief or other helpful interventions. When the need for orientation and control is satisfied by the maintenance of a delusional belief, MOTR would suggest that the therapist helps the patient to develop an intrinsic motivation to challenge his beliefs (e.g., with a four-field-schema).

### Negative Symptoms

The effects of pharmacological and psychotherapeutic interventions on negative symptoms are small (Elis et al., 2013; Chue and Lalonde, 2014). Is MOTR for schizophrenia able to address these treatment difficulties? First, using MOTR could support therapists to maintain a good therapy relationship even in face of severe negative symptoms such as blunted affect or alogia. When there is only minimal non-verbal or verbal feedback from the patient, therapists have an even higher demand for framework that guides therapeutic action in order to meet the needs of a patient, with MOTR being such a framework. Second, if future research should reveal that negative symptoms also have an instrumental aspect, MOTR might help to satisfy the motivational basis of negative symptoms.

### Summary

MOTR has a potential to guide therapists to find an unproblematic way to deal with problematic patient behavior and to optimize the conditions for effective psychological and pharmacological interventions for schizophrenia. A prerequisite for MOTR is an understanding of the individual motives of each patient. If therapists have no concept of the individual structure of motives, they are not able to use MOTR. Plan Analysis offers a framework for developing such an understanding (Caspar, 1995, 2007) and will be outlined in the next section.

## Inferring the Instrumentality of Experience and Behavior Using Plan Analysis

**FIGURE 1:**
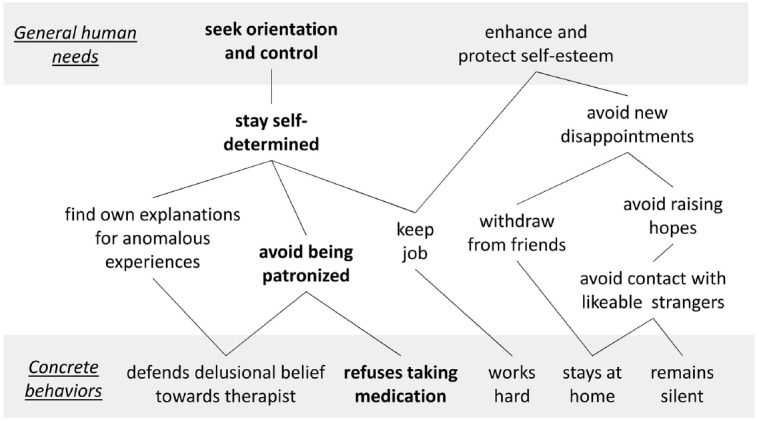
**A hypothetical Plan structure**.

The concept of Plan Analysis provides a psychological framework that helps to capture the instrumentality of problematic and unproblematic treatment-related behavior for superordinate motives (Caspar, 1995, 2007). The basic units of Plan Analysis are Plans^1^ which consist of a goal or purpose (e.g., exercise control) and a means (e.g., refuse medication). Plans are hierarchically nested within each other and can be graphically depicted as Plan structure, with basic human needs at the top and concrete behaviors at the bottom (for an example, see Figure 1). Grawe (2000) assumes four basic human needs: (1) orientation and control, (2) affiliation, (3) enhancement of self-esteem and (4) pleasure/avoidance of pain. *Approach* Plans motivate behavior which establishes congruent experiences (e.g., call friends → maintain close relationships), whereas *avoidance* Plans motivate behavior which prevents painful experiences (e.g., withdraw → avoid disappointments).

In the example in Figure 1, the patient works hard in order to keep his job. His Plan “keep job” serves to stay self-determined, and this Plan serves to maintain orientation and control. When this patient loses his job, for instance due to an exacerbation of schizophrenia, the Plan “keep job” is blocked. Under such circumstances, other Plans that serve the purpose of staying self-determined gain importance. In this case, the Plan structure is scarce regarding means for staying self-determined—that is, the Plan structure is *rigid*. The only remaining means for this patient is refusing to take medication in order to maintain his need for orientation and control. Besides rigidity of Plan structures, *multiple determination* is a prevalent property of Plans. In the example in Figure 1, the Plan “keep job” serves two superordinate Plans—staying self-determined and enhancing self-esteem. Losing the job, for instance because of schizophrenia, is accompanied by deprivation of *both* of the basic needs for autonomy and self-esteem, according to this Plan structure. In this view, problematic behaviors of patients, such as refusing medication, are goal-oriented and instrumental for satisfying their needs (e.g., staying self-determined), even though they might (as a negative side effect) undermine therapy outcome—at least unless the therapeutic offer takes them into account. It is crucial to infer the Plan structure of each patient individually, because the means which patients develop and use to satisfy their common basic needs can differ tremendously. Similarities within a group of patients—to which the term *prototypical Plans* refers—can nevertheless speed up the process of inferring Plans, but it needs to be plausible that a commonly found Plan makes sense also to this individual patient.

How to infer Plans? Patient reports are a valuable but not an exclusive source of information for hypotheses about Plans, particularly in patient with schizophrenia with reduced introspective and neuropsychological abilities (e.g., metacognitive capacities; Lysaker and Dimaggio, 2014). The use of multiple sources of information is highly important. In particular, there is a heavy weight on direct observation, especially of non-verbal behavior. Although hypotheses are constructed with the intention of coming as close as possible to patients’ actual Plans, one has to keep in mind that they are constructions rather than a reality. Helpful rules for inferring Plans are “always grounding hypotheses in multiple evidence” and “continually revising hypotheses based on new information” (see Caspar, 2011 for details).

### Emotions and Plan Analysis

From a Plan Analysis perspective, negative emotions signal that important Plans and basic needs are threatened or blocked, positive emotions that they are favored (Caspar, 2011) and are valuable diagnostic information. In general, five types of sources of emotions are assumed in Plan Analysis, which are described and illustrated in Table 1.

**TABLE 1:** **Sources of negative emotions from a Plan Analysis perspective**.

**Type**	**Description**	**Example**
Change in environment	The Plan structure, that is the totality of means for satisfying basic needs, does not fit to changes in the environment or to a new environment	An individual with first-episode psychosis is not able to satisfy his need for autonomy within the restricted setting of a secure ward with his usual Plan “Decide on appointments for yourself,” due to a predefined weekly schedule
Loss of individual abilities	The means for a purpose (e.g., skills) are no longer available	Neuropsychological deficits accompanying schizophrenia impede studying and block the Plan “graduate”
Rigid Plan structure	An important Plan has only a single (or too few) means for its realization	The Plan “Heighten self-esteem” is exclusively realized with the means “Stick to conviction of being loved by Jodie Foster” (erotomanic delusion)
Conflicting Plans	The means of a Plan endangers another important Plan	The Plan “Conceal hearing voices” endangers the Plan “Seek help when distressed”
Dominance of avoidance Plans	A high number of avoidance Plans reduce the degrees of freedom for realizing approach plans	The Plans “Avoid stress” and “Avoid a new psychotic episode” hinder the approach Plan “Try to make new friends”

Schizophrenia is often accompanied by a loss of individual abilities and changes in the environment (i.e., hospitalization, job loss, etc.). Under such circumstances, the flexibility and resilience of the patient’s Plan structure is particularly important. The more the structure is rigid or includes conflicting Plans, the more patients have problems satisfying their basic needs and experience negative emotions (see Table 1). Then, Plans might be in effect even though they have severe short- or long-term side effects, such as endangering the therapy relationship or even threating life (when a patient kills himself as a last demonstration of autonomy). For example, a loss of individual abilities might block most of the Plans related to self-esteem. Under such circumstances, the remaining Plans such as “maintain paranoid delusion” are particularly important for the patient, even though they can severely disturb the therapy relationship, highlighting the utility of MOTR for many patients with schizophrenia.

Although an individual Plan Analysis for each patient is necessary, findings from basic schizophrenia research should be taken into account to inform individual Plan Analyses were appropriate. In the next section, the instrumental perspective of Plan Analysis is used to review the literature of psychological mechanisms of the development and maintenance of schizophrenia.

### Need for Orientation and Control

Epstein (1990) assumed that there is a basic human need for orientation and control that helps individuals to make sense of their experiences and informs them about the degree of control they have in an environment. A deprived need for orientation and control is often signaled by anxiety. Orientation and control are psychological processes that are central to various models and findings from basic schizophrenia research and will be discussed in the next paragraph.

#### Cognitive Model of Psychosis

The cognitive model of positive symptoms of schizophrenia by Garety et al. (2001) as well as the more symptom-specific cognitive model of persecutory delusions by Freeman et al. (2002) or the cognitive model of auditory hallucinations (Mawson et al., 2010) propose that anomalous experiences or unspecific arousal motivate a search for an explanation or “meaning” (i.e., an appraisal process). The search for meaning in the cognitive models is influenced by emotional processes and cognitive biases. When individuals select a “threat belief” due to their search for meaning, a persecutory delusion develops (Freeman et al., 2002). The cognitive models stimulated further research and are also used for case formulations in CBTp (for a review, see Freeman and Garety, 2014). From an instrumental perspective, the key process of the models—“search for meaning”—is a Plan that serves the basic need for orientation and control. Accordingly, each attempt to challenge a delusional belief that serves the motive for orientation and control implies the risk of threatening an important Plan. Without offering an alternative explanation that is compatible to the Plan structure of a patient at first, this challenge is likely to result in anxiety, in attempts to protect the belief and in an alliance rupture.

#### Cognitive Biases

Cognitive biases such as jumping to conclusions (Fine et al., 2007) or bias against disconfirmatory evidence (BADE; Moritz and Woodward, 2006) influence the selection of an explanation for anomalous experiences, according to the cognitive models of psychosis (Garety et al., 2001). From a Plan Analysis perspective, cognitive biases might not solely express neuropsychological deficits but also be instrumental for satisfying the need for orientation and control and for avoiding conflictual views, being confronted with overdemanding complexity, and more.

#### Anxiety

Mounting evidence suggests that negative emotions play a central and causal role in the development and maintenance of positive symptoms such as delusions and acoustic hallucinations (Hartley et al., 2013; Marwaha et al., 2014). Studies with intensive longitudinal assessments revealed that increases in anxiety can trigger paranoid ideation in patients with schizophrenia (Thewissen et al., 2011). This effect is corroborated by experimental studies in sub-clinical populations, which suggest that anxiety can trigger paranoia (Lincoln et al., 2010; Westermann and Lincoln, 2010). Indirect evidence for the causal role of emotions in the development and maintenance of schizophrenia comes from pilot interventions studies that targeted worrying (Foster et al., 2010) and insomnia (Myers et al., 2011) without focusing on psychotic symptoms, but nevertheless reduced psychotic symptom severity. Taken together, the increase of anxiety is likely to trigger psychotic symptoms.

From a Plan Analysis perspective, anxiety might reflect a threatened or blocked need for orientation and control. Plans that increase orientation or facilitate control are expected to be especially relevant for experience and behavior in a state of anxiety, even if they are not adaptive on the long run. These Plans might include excessive worrying, acceptance of delusion-like spontaneous interpretations, retaining existing delusional interpretations that offer orientation and cognitive biases (e.g., jumping to conclusions). Moreover, helpful Plans which could serve as resources such as sleeping in order to prospectively regulate one’s emotions (Westermann et al., 2013) might be blocked.

### Need for Enhancing and Protecting Self-Esteem

Findings from basic clinical and psychotherapy research highlight the relevance of self-esteem for positive symptoms and therapeutic alliance in schizophrenia. Decreases in self-esteem can precede paranoid ideation in daily life according to studies with intensive longitudinal assessments (Thewissen et al., 2011). In experimental studies with sub-clinical samples, the effect of social exclusion on paranoid ideation was mediated by decreases in self-esteem (Kesting et al., 2013) and paranoid interpretations of social exclusion protected the self-esteem on a short-term scale (Lincoln et al., 2014). Lack of insight is accompanied by *less* self-stigmatization and demoralization across one year in patients with schizophrenia (Cavelti et al., 2014), with stigmatization being associated with lower self-esteem (Link et al., 2001). There is also evidence for a positive association of patient’s rating of the therapy relationship and their level of self-esteem in group interventions (Lecomte et al., 2012). Finally, prototypical Plans such as “Repair the self-esteem” were present in six of seven patients with schizophrenia in a qualitative study (Hellener, 1997).

Blocked or threatened Plans that serve to satisfy the need for self-esteem enhancement might be relevant for many individual Plan structures. In particular, rigid Plan structures (only limited means for enhancing self-esteem), conflicting Plans (the means of one motive such as affiliation block the means of another motive such as self-esteem) and a predominance of avoidance Plans (protection of self-esteem limits the opportunities for increasing self-esteem) are likely to be part of individual Plan structures of many patients with psychotic disorders. For example, the satisfaction of the need for self-esteem enhancement can depend on a rigid Plan structure of a patient, which includes solely the Plans “keep job” and “maintain belief of being loved by Jodie Foster.” When this patient is hospitalized, the Plan “keep job” is blocked. Under such circumstances, other sources for self-esteem enhancement should be established (e.g., validation by the therapist, social skills training, etc.; compare to Gassmann and Grawe, 2006) prior to a challenging the maintenance of the delusional belief, which is the very last means for protecting self-esteem.

### Other Basic Human Needs

#### Need for Affiliation

Attachment insecurity is associated with positive and negative symptoms with a small to medium effect size (Gumley et al., 2014). In line with this, attachment avoidance is accompanied by lower therapeutic alliance (Berry et al., 2007) and insecure attachment is a risk factor for disengagement from mental health services (Tait et al., 2004). Thus, Plan Analysis with patients with schizophrenia should focus on attachment related Plans in detail.

#### Need for Pleasure/Avoidance of Pain

Mounting evidence suggest that emotion regulation difficulties are related to psychotic symptoms (e.g., Westermann and Lincoln, 2011) and schizophrenia in general (O’Driscoll et al., 2014). For instance, the generally adaptive emotion regulation strategy cognitive reappraisal is less frequently used by patients with schizophrenia compared to controls (O’Driscoll et al., 2014), and findings from a study with sub-clinical samples suggest that reappraisal might be even maladaptive for delusion-prone individuals under social stress (Westermann et al., 2012). In addition, Owens et al. (2013) report a negative correlation between the therapy relationship and difficulties in emotion regulation in patients with schizophrenia and related disorders. Thus, demands in the therapeutic relationship should not threaten emotion regulation strategies unless alternatives to them can be offered or developed. The inclusion of Plans for emotion regulation that serve the basic need for pleasure and avoidance of pain might be important in individual Plan structures.

It needs to be emphasized that, although plausible in a particular frame of reference, Grawe’s four “basic needs” are not exclusive at all. When truly inferring in an inductive bottom-up manner, one finds different needs with different individuals. For example, seeking for meaning appears for many patients as an independent need which goes far beyond seeking orientation, as outlined above.

## Summary and Outlook

The assumption that unproblematic and problematic behaviors are instrumental for satisfying the needs of a person is the basis for MOTR. A good therapy relationship can be built and maintained by (1) being responsive and proactively satisfying regarding the important *unproblematic*, acceptable patient behaviors and Plans and (2) being unresponsive to *problematic* behaviors but responsive and proactively satisfying regarding the superordinate acceptable motives to which problematic behaviors hypothetically serve. A prerequisite for MOTR is an understanding of individual motives of a patient, for instance by using Plan Analysis. Plan Analysis has to be carefully and continuously conducted for each patient and can include and integrate findings from basic clinical research if appropriate for a specific patient. Findings from basic research suggest that many patients with schizophrenia have suboptimal Plan structures with regard to autonomy and self-esteem. When a patients’ Plan structure does not fit to a new environment or a loss of individual abilities or both (as it seems often to be the case in schizophrenia), patients make frequent and intensive use of problematic behaviors that endangers the therapy relationship due to a lack of alternative Plans. MOTR can guide therapists to build and maintain a good therapy relationship despite such problematic behaviors.

### Implications for Psychotherapy

The main advantage of MOTR in schizophrenia treatments is expected to be that evidence-based interventions from different fields such as pharmacotherapy, CBT or family therapy can be applied more effectively. However, Plan Analysis has more potential than only guiding in-session therapist behavior. Plan Analysis can enrich a case formulation and inform a therapist about patient resources, appropriate skills trainings, and the reasonable sequence of interventions. For example, if the Plan “maintain belief that the voice comes from an angel” serves the need for affiliation and self-esteem, and the patient has only few other means for satisfying these needs, one should include interventions for building up affiliation resources and self-esteem enhancement in the treatment plan. An accordingly staged treatment plan could be: (1) activating and validating patient resources in order to build up a therapy relationship, (2) skills training for enriching the rigid Plan structure for self-esteem and affiliation (e.g., social skills training) and cognitive disputation of automatic thoughts that reflect conflicting Plans, (3) cognitive disputation of the auditory hallucination related delusions (if they are still maintained), and (4) relapse prevention.

Currently, more and more specific interventions for schizophrenia are being developed and evaluated. These interventions target emotion-related processes such as worrying or insomnia, are directly derived from basic research and are a valuable contribution to the treatment of schizophrenia (Foster et al., 2010; Myers et al., 2011). However, not all “negative” emotions stem from psychopathological processes such as worrying or sleep deprivation due to insomnia. In order to understand the generation of emotions that might trigger psychotic symptoms in a more generic manner, one can make use of Plan Analysis, because it captures recurring emotions on a broader scope. For example, if a patient with schizophrenia experiences negative emotions due to Plans related to a comorbid social phobia, the therapist can address this source of distress by offering the patient exposure therapy (without having to wait on the first treatment manual for exposure therapy in patients with schizophrenia with comorbid social phobia).

### Implications for Pharmacotherapy

When psychiatrists are able to identify their patient’s needs and to adapt to them, the instrumentality of the problem behavior “refusal of antipsychotic medication” is less likely to be overlooked. Vicious circles of problematic patient and problematic therapist behaviors could be prevented or stopped (e.g., therapist tries to coerce patient to being insightful in order to take medication, patient refuses medication in order to experience himself as autonomous, and so on). If future empirical research corroborates the hypothesis that there are prototypical Plan structures for patients with schizophrenia, short MOTR trainings for psychiatrists would be feasible and beneficial that do not require psychiatrist to conduct a complete Plan Analysis for each patient.

### Research Implications

A research agenda for MOTR for schizophrenia encompasses three main domains. First, the hypothesis that motivational constructs such as blocked or threatened Plans are relevant to the therapeutic relationship in addition to other variables such as symptom severity or neuropsychological deficits has to be tested. Second, individual Plan Analyses of patients with schizophrenia are necessary in order to empirically determine the validity and reliability of Plan Analysis in schizophrenia. Possibly, such studies will reveal prototypical Plan structures that help to inform future research and trainings. Third, randomized controlled trials are necessary to test whether treatments as usual (e.g., pharmacotherapy, CBTp, etc.) enriched with MOTR are more effective than treatments as usual without MOTR. In addition, such intervention studies would investigate whether therapists that adopt MOTR with patients with schizophrenia are more comfortable and experience themselves as less helpless when using MOTR, because they are able to see the underlying acceptable human needs of their patients’ problematic behaviors and can thus avoid vicious circles of problematic behaviors (see Schmitt et al., 2003). Thus, MOTR has the potential to contribute to the mental health of therapists and other care providers.

Taken together, MOTR has the potential to improve both pharmacological and non-pharmacological treatment for schizophrenia by enabling therapists to proactively adapt to their patient’s motives and to preventing problematic behavior. The approach makes use of (current and future) findings from basic and psychotherapy research and is generic in this respect, but at the same time guides therapeutic action precisely and flexibly in a patient oriented way.

### Conflict of Interest Statement

The authors declare that the research was conducted in the absence of any commercial or financial relationships that could be construed as a potential conflict of interest.

## Footnotes

1 Written in the upper case following the tradition of (Miller et al., 1960) serving to highlight that their use of Plans differs from the common use, mainly in that they consider most Plans to be non-conscious.
